# The roles of trimethylamine-N-oxide in atherosclerosis and its potential therapeutic aspect: A literature review

**DOI:** 10.17305/bb.2023.8893

**Published:** 2023-12-01

**Authors:** Yudi Her Oktaviono, Ariikah Dyah Lamara, Pandit Bagus Tri Saputra, Jannatin Nisa Arnindita, Diar Pasahari, Mahendra Eko Saputra, Ni Made Adnya Suasti

**Affiliations:** 1 Department of Cardiology and Vascular Medicine, General Hospital Dr. Soetomo, Faculty of Medicine, Universitas Airlangga, Surabaya, East Java, Indonesia; 2 Faculty of Medicine, Universitas Airlangga, Surabaya, East Java, Indonesia; 3 Nuffield Department of Medicine, University of Oxford, Oxford, United Kingdom

**Keywords:** Atherosclerosis, gut microbiome (GM), trimethylamine-N-oxide (TMAO), coronary artery disease, cardiovascular risk

## Abstract

Current research supports the evidence that the gut microbiome (GM), which consists of gut microbiota and their biologically active metabolites, is associated with atherosclerosis development. Trimethylamine-N-oxide (TMAO), a metabolite produced by the GM through trimethylamine (TMA) oxidation, significantly enhances the formation and vulnerability of atherosclerotic plaques. TMAO promotes inflammation and oxidative stress in endothelial cells, leading to vascular dysfunction and plaque formation. Dimethyl-1-butanol (DMB), iodomethylcholine (IMC), and fluoromethylcholine (FMC) have been recognized for their ability to reduce plasma TMAO by inhibiting TMA lyase, a bacterial enzyme involved in the choline cleavage anaerobic process, thus reducing TMA formation. Conversely, indole-3-carbinol (I3C) and trigonelline inhibit TMA oxidation by inhibiting flavin-containing monooxygenase-3 (FMO3), resulting in reduced plasma TMAO. The combined use of inhibitors of choline TMA lyase and FMO3 could provide novel therapeutic strategies for cardiovascular disease prevention by stabilizing existing atherosclerotic plaques. This review aims to present the current evidence of the roles of TMA/TMAO in atherosclerosis as well as its potential therapeutic prevention aspects.

## Introduction

Cardiovascular disease (CVD) is the leading cause of morbidity and mortality worldwide. The prevalence of atherosclerosis, identified as the underlying pathophysiology of CVD, is increasing worldwide despite efforts to address the risk factors, such as smoking, hypertension, hyperlipidemia, diabetes, and obesity [1]. The pathophysiology of atherosclerosis begins with endothelial dysfunction associated with a cascade of events, such as lipid accumulation, calcification, and fibrous cap elements that will lead to a narrowed blood vessel passage and trigger an inflammatory response [1, 2]. Researchers discovered bacterial DNA in atherosclerotic plaques, suggesting a possible link between the features of atherosclerosis and the role of gut microbes and their biologically active metabolites in CVD. Moreover, increased vascular endothelial dysfunction [2–4], facilitated thrombosis [5], and increased plaque instability [6] correlate with plasma trimethylamine-N-oxide (TMAO), the trimethylamine (TMA) oxidation product of gut microbiota, and TMAO which is present in animal-based food (e.g., egg, fish, red meat, and poultry). It has been found that TMAO promotes the formation of atherosclerosis through several mechanisms, including decreased bile acid biosynthesis, alteration of reverse cholesterol transport (RCT), foam cell formation, and accumulation of cholesterol in macrophages [2–6].

The bacterial enzyme TMA lyase, involved in the anaerobic choline cleavage, is inhibited by 3,3-dimethyl-1-butanol (DMB), resulting in reduced TMA production and a decrease in TMAO formation. At the same time, indole-3-carbinol (I3C) reduces TMAO formation in plasma by inhibiting flavin-containing monooxygenase-3 (FMO3) for TMA oxidation [7]. In recent years, several studies have provided evidence that the gut microbiome (GM) is associated with the development of atherosclerosis. However, the relationship between the GM and plaque instability remains unclear. Therefore, this review aims to provide current evidence for the role of TMAO, one of the GM metabolites, in the development of atherosclerosis and plaque instability. We also discuss combinations of TMA/TMAO formation inhibitors as possible therapeutic prevention aspects.

## Gut microbiota in atherosclerosis

The “gut microbiota” comprises the diversity of bacteria found in the human gastrointestinal (GI) system, preferably in the ascending colon, which is a nutrient-rich anaerobic environment [5]. In contrast, the “GM” is the symbiotic relationship between gut microbes and the potent biologically active metabolites they produce [1]. Alterations in the structure and function of the microbial population are associated with various diseases, including CVD [8]. A “dysbiosis” which is an alteration and/or imbalance of microbial populations, is usually the beginning of the pathophysiology of diseases [1]. In adults, the gut microbiota is mainly composed of five phyla: *Bacteroidetes* and *Firmicutes* (90% of all bacterial species), *Actinobacteria*, *Proteobacteria*, and *Cerrucomicrobia*. The Firmicutes/Bacteroidetes ratio, commonly referred to as the F/B ratio, may be a health parameter of the gut microbiota composition, although it is not always the same in different individuals. The ratio shifts in the elderly suggest changes related to the occurrence of age-related CVD [1, 9]. An increase in the F/B ratio is equivalent to an increase in the likelihood of plaque rupture, and thus CVD events [9]. The concept of “leaky gut” is a dysfunction of the functional mucosal barrier, which includes elements critical for maintaining balanced symbioses, such as mucus production, the tight junctions between epithelial cells, and mucosal immunology [9]. Gut dysbiosis may also be due to alterations in host barrier function, resulting in damaged gut permeability [5]. Overall, leaky gut results in the translocation of bacteria in the host bloodstream, as well as certain microbial contaminants, leading to an inflammatory response, which further sets off several aberrant immunological responses and the onset of atherosclerosis [10].

Several studies have confirmed the presence of bacterial DNA in atherosclerotic plaques, reinforcing the correlation between the microbiota and the development of CVD. This is substantiated by research that has shown differences in the gut microbiota between individuals with atherosclerosis and those without the condition. In specific studies, patients with coronary heart disease or high intima media thickness (IMT) values exhibited a higher F/B ratio, a characteristic typically observed in obese individuals. Additionally, some researchers have found that bacterial DNA can provoke macrophages to activate the innate immune system. This process involves toll-like receptor (TLR) 2 and TLR4, which contribute to the stability of atherosclerotic plaques [1, 9].

**Figure 1.**
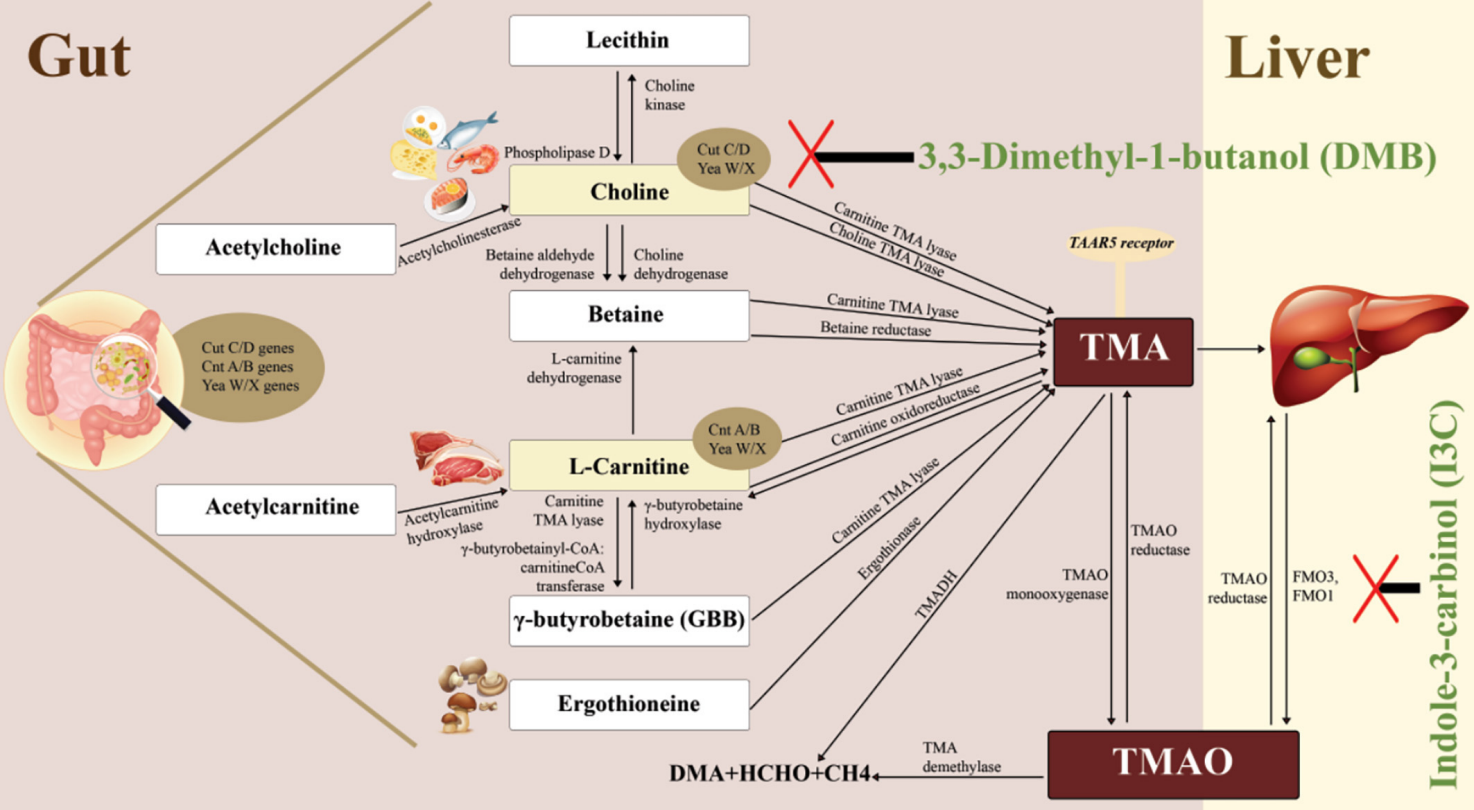
**Various TMAO mechanisms leading to atherosclerotic plaque formation and instability in the gut.** The gut microbiome mainly contains cutC/D gene, cntA/B gene, and yeaW/X gene containing bacteria, which are involved in the human digestive system by producing various enzymes. Various nutrient precursors (such as choline, lecithin, betaine, carnitine, and ergothioneine), which are mostly present in animal-derived products, are processed by the gut microbiota through enzymatic processes, producing TMA that specifically interacts with the TAAR5 receptor. TMA is then converted to the proatherogenic TMAO mostly by hepatic FMO3 enzymes, followed by hepatic FMO1 and intestinal TMAO monooxygenase. DMB can inhibit the cutC/D gene TMA lyase activity resulting in decreased formation of TMA, while I3C inhibits hepatic FMO3 and FMO1 enzymes, resulting in lower formation of TMAO. TMAO: Trimethylamine-N-oxide; cutC/D: Cut gene cluster genes C and D; cntA/B: Cnt gene cluster genes A and B; yeaW/Y: Yea gene cluster genes W and Y; TMA: Trymethylamine; TAAR5: Trace amine associated receptor 5; FMO3: Flavin-containing monooxygenase 3; FMO1: Flavin-containing monooxygenase 1; DMB: 3,3-dimethyl-1-butanol; I3C: Indole-3-carbinol; CoA: Coenzyme A; TMADH: Trimethylamine dehydrogenase; DMA: Dimethylamine; HCHO: Methanol; CH4: Methane.

The gut microbiota primarily produces a significant number of metabolites, one of which is TMAO. This conversion product, found in animal-based foods, contributes directly to the pathogenesis of atherosclerosis. TMAO originates from several nutrients, including L-carnitine, choline, and betaine, all of which are derived from the gut microbiota species Firmicutes. These nutrients are later converted into TMA, which is absorbed into the bloodstream. Subsequently, it is processed in the liver and transformed into TMAO by the enzyme FMO3. These complete mechanisms are shown on Figure 1. Under normal circumstances, these metabolites are excreted by the kidneys through urine, accounting for approximately 95% of excretion. However, any alterations in the hepato-renal system can influence this process and result in an elevated TMAO level, a condition linked to atherosclerosis [1, 6, 9].

## TMA/TMAO effect on atherosclerosis formation and plaque instability

### TMAO—biogenesis and metabolism

TMAO is an active metabolite produced by gut microorganisms through the oxidation of TMA. The primary nutrient precursors of TMA mainly contain free choline and choline-containing compounds like phosphatidylcholine (lecithin), phosphocholine, glycerophosphate-choline, sphingomyelin, as well as L-carnitine, betaine, or betaine-containing compounds (γ-butyrobetaine [GBB], cronobetaine, and trans-crotonobetaine), ergothioneine, and trimethyl-lysine. TMA is abundant in an omnivorous diet that includes animal products, such as salt-water fish, eggs, red meat, and dairy products. Additionally, fish is known to have high levels of TMAO, which is suspected to be a contributing factor to the prevalence of atherosclerosis in the Japanese population [10].

These nutrient precursors are transformed into TMA, an odorous pro-atherogenic metabolite gas, by enzymes encoded by gut microorganisms, primarily belonging to the *Enterobacteriaceae* family. These enzymes can be categorized into three types: (1) microbial cutC/D (cut gene cluster genes C [catalytic] and D [activating]) enzymes, which produce choline TMA-lyase and are found in *Desulforibrio*, *Desulfuricans*, *Proteobacteria* and *Proteus mirabilis*, and *Firmicutes* (*Clostridia*, *Bacillus*); (2) cntA/B (cnt gene cluster genes A [oxygenase] and B [reductase]) enzymes, which act as carnitine monooxygenases and are primarily produced by *Acinetobacter* and *Proteobacteria*; (3) yeaW/X (yea gene cluster genes W [oxygenase] and X [reductase]) enzymes, found in *γ-Proteobacteria* species, which specifically utilize various substrates like betaine, GBB, and lecithin to generate TMA [11, 12].

Choline TMA-lyase binds to choline through aromatic hydrogen bonding at the Phe677, Cys771, and Glu773 sites. During an anaerobic process, it cleaves dietary choline by breaking its CN bonds, resulting in the production of TMA and acetaldehyde as by-products [13]. Betaine, primarily found in plants, can undergo a reduction–oxidation process catalyzed by betaine reductase to convert into TMA. Top of form choline can also be converted into betaine by two enzymes that act in sequence (choline dehydrogenase and betaine aldehyde dehydrogenase) [14]. L-carnitine, which is the L-stereoisomer found in animal products, can be primarily converted to TMA by carnitine oxidoreductase. Additionally, L-carnitine can be transformed into GBB and betaine through the action of two enzymes: GBB-butyrobetainyl-CoA carnitine CoA transferase and L-carnitine dehydrogenase, respectively. There is another enzyme called carnitine TMA lyase that can convert GBB, choline, betaine, and carnitine into TMA. GBB, in turn, can be converted back into L-carnitine by the enzyme butyrobetaine hydroxylase [15].

TMA can also be produced by the metabolism of ergothioneine, a biogenic amine found in mushrooms, beans, and animal parts specifically kidneys and liver [15]. When TMAO is consumed, approximately half of it is absorbed and excreted intact in the urine. The remaining portion is converted into TMA by intraluminal TMAO reductase. TMA monooxygenase breaks down intraluminal TMA to TMAO. Furthermore, methanogenic bacteria that contain the enzymes TMA dehydrogenase (TMADH) and TMAO demethylase may deplete TMA and TMAO, resulting in the production of dimethylamine (DMA), formaldehyde, ammonia, and methane [16]. TMA is absorbed into the portal vein circulation through passive diffusion across enterocyte membranes, transferred to the liver, and rapidly converted into TMAO by host hepatic FMOs, primarily FMO3 and FMO1. FMO1 exhibits ten times less specific activity in the liver compared to FMO3. Furthermore, some TMA is oxidized in the intestines (jejunum and cecum) by TMA monooxygenase [9, 17, 18]. TMA/TMAO from food follows the same metabolic pathway as its putative precursors. The majority of TMA/TMAO has a high turnover rate and a quick clearance rate through renal excretion. Within 24 h, approximately 95% of TMA is oxidized and excreted in the urine in a 3:95 ratio of TMA to TMAO. Only 4% of TMA is lost in feces, and less than 1% is eliminated in the breath [17]. Normal plasma TMAO levels are approximately 0.55 µM, while the plasma TMAO cut-off value is > 6 µM. Elevated plasma TMAO levels are associated with an increased risk of malignant CVD events. An increase in TMAO plasma levels could lead to a 74% risk of major adverse cardiac events (MACE) and a 66% increase in all-cause mortality [19]. For every 10-µmol/L increase in TMAO, there is an 8% higher incidence of stroke [20], a 20% risk increase in hypertension [21], and a 7.6% rise in overall mortality [9, 18, 22].

### TMAO—inflammation and oxidative stress

Atherosclerosis is a chronic inflammatory disease that results from multiple mechanisms of plaque formation inside the blood vessels. It is caused by impaired lipid homeostasis, impaired cholesterol metabolism, impaired cholesterol transport, endothelial dysfunction, vascular inflammation, and macrophage foam cell formation which, in combination with plaque rupture and increased platelet reactivity, lead to thrombus formation that narrows the vascular passage and decreases blood flow, potentially leading to vascular embolism [23]. Atherosclerotic plaques consist of a lipid-rich atheromatous core covered by a collagen-rich fibrous cap. The compiled lipid-rich atheromatous component weakens the fibrous cap and causes further inflammation of the thrombogenic internal arterial wall, making the plaque more prone to rupture [24].

Even when accounting for traditional risk factors, the plasma levels of TMAO and the nutrient precursors containing TMA show a correlation with accelerated atherosclerosis, increased platelet reactivity, and heightened potential for thrombosis. These associations are attributed to mechanisms dependent on gut microbiota, although the specific underlying mechanism is yet to be fully understood [5, 9, 25]. The elevated levels of TMAO contribute to dysfunction in lipid-cholesterol-bile acid metabolism, oxidative stress, vascular inflammation, upregulation of adhesion molecules, recruitment of immune cells (primarily leukocytes), and platelet aggregation.

Some studies have shown that higher plasma TMAO levels are closely related to coronary atherosclerotic plaque size and plaque rupture, as well as non-culprit plaques. Several vulnerable characteristics found were thinner fibrous cap thickness, thin cap fibroatheroma plaques with at least two quadrants of lipid component, and fibrous cap thickness of ≤65 µm, and the presence of immature microvessels causing an imbalance between anti-angiogenic and pro-angiogenic factors, thereby simultaneously increasing intraplaque hemorrhage [26].

TMAO has been reported to be involved in the activation of heat shock proteins (HSP-70 and HSP-60) and glucose-regulated proteins (GRP-94 and GRP-78), which are critical for the initial inflammatory step of atherosclerosis. It is involved in the pro-inflammatory development of foam cells through the Toll-like receptors (TLR-4 and TLR-2) [27, 28], which can also be activated by macrophage scavenger receptors (scavenger receptor A1 [SR-A1] and the cluster of differentiation 36 [CD-36]).

TMAO increases pro-inflammatory CD14U+, CD16+, patrolling lymphocyte antigen 6 complex locus C (Ly6C) monocytes [29] and induces the apoptosis-associated speck-like protein containing caspase activation and recruitment domain (ASC) activation into nucleotide oligomerization domain (NOD)-like receptors (NLRs) 3 (NLRP3). The NLRP3 inflammasome regulates cell death processes (pyroptosis, apoptosis, and necroptosis) to maintain host homeostasis.

TMAO promotes the expression of inflammatory mediators, such as caspase-1, interleukin (IL)-1β, IL-6, and IL-18 [30] and triggers the mitogen-activated protein kinase (MAPK) and nuclear factor kappa light chain enhancer of activated B cells (NF-κB) signaling pathway. All of this further increases the expression of tumor necrosis factor alpha (TNF-α), C-reactive protein (CRP), and IL-1 [31].

MAPKs targeting the c-jun N-terminal kinase (JNK), extracellular signal-regulated kinase (ERK), and p38 MAPK protein play key roles in controlling the response of cells to diverse stimuli, including heat shock and proinflammatory cytokines. Multiple biological activities, including cell proliferation, differentiation, and migration, critically depend on the ERK cascade. JNK is involved in the regulation of several cellular processes, including apoptosis and proliferation. The p38 MAPK signaling pathway enables cells to recognize various environmental signals. Overall, MAPK signaling pathways may contribute to the development of atherosclerosis by controlling the division and migration of vascular endothelial cells [32]. NF-κB is an inducible transcription factor that controls genes involved in the initiation and progression of inflammation [33]. In addition to the immune-related classical inflammatory signaling pathway, TMAO also influences oxidative stress-related inflammation. As a result, decreased synthesis of nitric oxide (NO) and endothelial-derived NO synthase (eNOS) expression and increased reactive oxygen species (ROS) negatively affect the maintenance of normal vascular function [34]. It activates the inflammatory signaling pathway involving mitochondrial ROS production and the binding of thioredoxin interacting protein (TXNIP) with thioredoxin (TXR-1 and TXR-2, with TXR-2 being mitochondrial specific), forming the mitochondrial ROS-TXNIP-NLRP3 signaling pathway. This pathway promotes the release of IL-1β and IL-18 in a dose and time-dependent manner and promotes the production of pro-caspase-1 cleavage into caspase-1. It increases oxidative stress, decreases antioxidant defenses, disrupts the endothelial function, and releases pro-inflammatory cytokines [35, 36]. The pathway also inhibits the nicotinamide adenine dinucleotide (NAD)-dependent deacetylase sirtuin-3 (SIRT3)–superoxide dismutase-2 (SOD2)–mitochondrial antioxidant pathway, thereby increasing inflammatory cytokines, suppressing eNOS expression and NO synthesis and accelerating the onset and progression of endothelial damage [37]. In addition, at elevated plasma TMAO levels, a dose-dependent suppression of the autophagy-related 16 like 1 (*ATG16L1*) gene expression, a protein required for the initial stages of autophagy, is observed. It reduces the amount of autophagy as a mechanism for maintaining cell survival and homeostasis that may protect against atherosclerosis [38]. The exact mechanisms by which TMAO triggers inflammasome activation are still unknown.

### TMAO—cholesterol metabolism and foam cell formation

TMAO can dysregulate the mechanisms of vasorelaxation by increasing the production of endothelin-1 (ET-1), causing endothelial dysfunction [6]. TMAO also increases blood levels of monocyte chemoattractant protein 1 (MCP-1), which supports monocyte recruitment and promotes inflammation and local lipid accumulation [39, 40].

The accumulated lipids in the arterial wall, consisting mainly of cholesterol, contribute to the pathophysiology of atherosclerosis. The four processes that cause cholesterol build-up in the arterial walls are: (1) decreased excretion of bile acids (BA), (2) increased cholesterol synthesis, (3) increased cholesterol influx into the arterial wall, and (4) decreased cholesterol efflux from the arterial wall. TMAO is involved in the accumulation of cholesterol by altering sterol metabolism in various compartments, including the BA compartment as the primary enterohepatic conversion and excretion of cholesterol in BA [41]. Cholesterol 27α-hydroxylase (CYP27A1) and cholesterol 7α-hydroxylase (CYP7A1) as the primary BA production enzymes are downregulated by TMAO through the activation of the small heterodimer partner (SHP) and farnesoid X receptor (FXR) nuclear receptors [42]. TMAO also reduces the size of the BA pool and thus the ability to remove cholesterol [41]. Furthermore, by stimulating the protein kinase R-like endoplasmic reticulum kinase (PERK) pathway and hepatic liver X receptor (LXR) signaling, which also regulates the endoplasmic reticulum stress (ERS) and inflammation, TMAO accelerates the worsening of atherosclerotic plaque formation, as one of the crucial roles of endoplasmic reticulum is in lipid biosynthesis.

TMAO interferes with cholesterol metabolism by disrupting the cholesterol transport mechanism called transintestinal cholesterol excretion (TICE), thereby increasing total cholesterol levels (TC), total circulating triglycerides (TG), very-low-density lipoprotein cholesterol (VLDL-c), and low-density lipoprotein cholesterol (LDL-c), while simultaneously lowering levels of high-density lipoprotein cholesterol (HDL-c) [36, 43]. The expression of the intestinal cholesterol transporters Niemann-Pick C1-like 1 (NPC1L1), which transports cholesterol into enterocytes, and the adenosine triphosphate (ATP)-binding cassette (ABC) G5 (ABCG5) and G8 (ABCG8), or as a combined ABCG5/8 transporter, which transport cholesterol out of enterocytes, were both found to be decreased by TMAO, resulting in decreased cholesterol absorption [20, 44]. All of these mechanisms mentioned above eventually impede the macrophage RCT, resulting in a pro-atherogenic effect. RCT is a process with which the macrophages prevent the accumulation of additional cholesterol and facilitate its efflux from foam cells deposited in the atherosclerotic intima. The cholesterol which is effluxed from the foam cells travels to the liver and the intestines for fecal excretion [45].

The characteristic initial stage of atherogenesis involves the formation of proatherogenic foam cells in the arterial intima, characterized by uncontrolled accumulation of lipoproteins due to impaired macrophage function. Macrophages regulate lipoprotein metabolism and transport and are the primary immune cells in atherosclerotic lesions. TMAO contributes to the degenerative phase of atherosclerosis development by promoting macrophage movement and further augments atherosclerotic lesions by increasing the expression of macrophage scavenger receptors CD-36 and the SR-A1, inducing foam cell formation and impairment of in vivo RCT [46]. Scavenger receptors contribute to the processing and regulation of modified lipoproteins by introducing extracellular elements inside the cells and designating them for lysosomal degradation leading to macrophage dysfunction, which in turn promotes foam cell formation.

In atherosclerosis, low-density lipoproteins (LDLs) in the arterial walls are often oxidatively converted to oxidized LDLs (ox-LDLs). After differentiating from monocytes through the actions of CD36 and SR-A1 scavenger receptors, macrophages can recognize and uptake ox-LDLs, thereby activating their expressions as well [31]. TMAO can enhance the expression of CD36 and SR-A1 scavenger receptors in macrophages, encouraging greater ox-LDL build-up and foam cell production. Moreover, similar to ox-LDL, TMAO acts in the opposite direction and promotes the development of monocytes into macrophages, which primarily scavenge ox-LDL, leading to macrophage foam formation [21]. The MAPK/ERK/JNK pathway appears to be critical for macrophage foaming, stimulating the production of pro-inflammatory cytokines and encouraging macrophage migration to the endothelial barrier, which contributes to the formation of atherosclerotic plaques in the form of foam cells [31].

By upregulating the SR-A1 receptors, TMAO also downregulates the ATP-binding cassette transporters A1 (ABCA1) and ATP-binding cassette transporters G1 (ABCG1) in macrophages, thus enhancing ERS. Higher expression of ABCA1 leads to downregulation of the homotetrameric enzyme ATP citrate lyase (Acly), an enzyme that catalyzes the formation of cytosolic acetyl coenzyme A (acetyl-CoA) and oxaloacetate from citrate, providing a substrate for the de novo production of fatty acids (lipogenesis) and cholesterol, while also indirectly controlling macrophage activation through histone acetylation. The lack of macrophage Acly stabilizes atherosclerotic plaques. As a result, by downregulating ABCA1 expression in macrophages, TMAO worsens the atherosclerosis [47].

Macrophages are known to be a diverse group of tissue-inhabiting professional phagocytes that evolved from THP-1 monocytes (human leukemia monocytic cell line), activated into two main polarization states, resulting in opposite phenotypic forms as the classically activated pro-inflammatory macrophages type 1 (M1) and alternatively activated anti-inflammatory macrophages type 2 (M2) cells. The production of pro-inflammatory cytokines, including TNF-α, interleukin 1 (IL-1), IL-6, and IL-12, is a feature of M1 macrophages, which also generate ROS, leading to oxidative stress that causes inflammation. Alternatively, activated macrophages can also be polarized into M2 macrophages by IL-4 and IL-13, which release anti-inflammatory cytokines such as IL-10. TMAO can cause macrophages to become polarized to M1 macrophages through the expression of NLRP3. Therefore, TMAO can exacerbate the development of atherosclerotic plaques by enhancing the inflammatory response of macrophages [48, 49]. Further research on the exact mechanism is needed.

### TMAO—platelet activation and thrombosis

Elevated plasma TMAO levels also reduce circulating human endothelial progenitor cells (EPCs) which are crucial for endothelial repair and participation in new artery formation. This is due to TMAO-induced upregulated expression of vascular cell adhesion molecule-1 (VCAM-1) by the protein kinase C (PKC)/NF-κB pathway, which facilitates the human monocytic THP-1 adhesion [41] and reduces endothelial self-repair [36]. PKC is an important enzyme family that regulates gene expression and cell proliferation. Moreover, TMAO levels cause the migration and adhesion of activated leukocytes with their recruitment to the vasculature, encouraging endothelial cell activation through the MAPK/NF-κB pathway [5]. E-selectin, VCAM-1 and intracellular adhesion molecule 1 (ICAM-1) are important adhesion molecules that not only accelerate atherosclerosis but also promote thrombosis [7].

**Figure 2.**
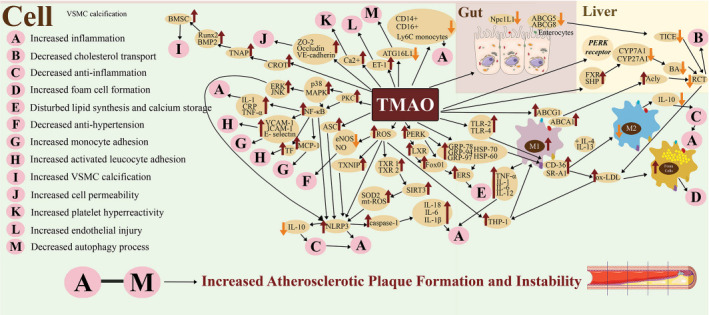
**Various TMAO mechanisms leading to atherosclerotic plaque formation and instability in the cell.** Elevated plasma TMAO levels, already circulating in the blood, trigger several processes leading to atherosclerotic plaque formation and instability. (A) TMAO increases inflammation by producing activated monocytes, increasing the CD14/16 expression and by increasing the pro-inflammatory cytokines by the M1, NLRP3 inflammasome, and NF-κB. The increased inflammation may also be caused by a lack of anti-inflammatory cytokines (IL-10). (B) TMAO disrupts RCT, resulting in impaired conversion of cholesterol into the BA and impaired cholesterol transport, which in turn leads to an increase in circulating total cholesterol and ox-LDL. (C) TMAO decreases the production of anti-inflammatory cytokines. (D) TMAO increases the formation of foam cells, which are critical for plaque formation. (E) TMAO interferes with lipid synthesis and calcium deposition by increasing ERS. (F) TMAO decreases the production of NO and eNOS expression, leading to endothelial dysfunction that causes hypertension. (G) TMAO increases monocyte adhesion by increasing the production of the adhesion protein MCP-1. (H) TMAO increases the activated leucocyte adhesion by increasing the production of the tissue factor. (I) TMAO may cause vascular calcification by triggering the protein responsible for VSMC. (J) TMAO increases cell permeability by decreasing proteins responsible for cell membrane maintenance. (K) TMAO increases platelet hyperreactivity by increasing Ca2+ signaling, which leads to platelet hyperreactivity to various signals, resulting in increased thrombus formation. (L) TMAO increases endothelial injury through the production of ET-1. (M) TMAO decreases the autophagy process, which is critical for maintaining endothelial cell stability. VSMC: Vascular smooth muscle cells; TMAO: Trymethyamine-N-oxide; PKC: Protein kinase C; p38: Protein 38; MAPK: Mitogen-activated protein kinase; ERK: Extracellular signal-regulated kinase; JNK: C-jun N-terminal kinase; NF-κB: Nuclear factor kappa light chain enhancer of activated B cells; IL: Interleukin; CRP: C-reactive protein; TNF-α: Tumor necrosis factor alpha; VCAM-1: Vascular cell adhesion molecule 1; ICAM-1: Intracellular adhesion molecule 1; MCP-1: Monocyte chemoattractant protein 1; TF: Tissue factor; NLRP3: Nucleotide oligomerization domain (NOD)-like receptors 3; ASC: Apoptosis-associated speck-like protein containing caspase activation and recruitment domain; ROS: Reactive oxygen species; eNOS: Endothelial-derived nitric oxide synthase; NO: Nitric exide; TXNIP: Thioredoxin interacting protein; TXR-1: Thioredoxin 1; TXR-2: Thioredoxin 2; SIRT3: Sirtuin 3; SOD2: Superoxide dismutase 2; mt-ROS: Mitochondrial reactive oxygen species; THP-1 monocyte: Human leukemia monocytic cell line; PERK: Protein kinase R-like endoplasmic reticulum kinase; LXR: Liver X receptor; Fox01: Fork-head box protein 01; ERS: Endoplasmic reticulum stress; GRP-78: Glucose regulated protein 78; GRP-94: Glucose regulated protein 94; GRP-97: Glucose regulated protein 97; HSP-60: Heat shock protein 60; HSP-70: Heat shock protein 70; TLR-2: Toll-like receptors 2; TLR-4: Toll-like receptors 4; M1: Macrophage 1; CD: Cluster of differentiation; SR-A1: Scavenger receptor A1; M2: Macrophage 2; ox-LDL: Oxidized low-density lipoprotein; ABCG1: Adenosine triphosphate (ATP)-binding cassette transporters G1; ABCA1: ATP-binding cassette transporters A1; Acly: ATP citrate lyase; RCT: Reverse cholesterol transport; FXR: Farnesoid-X-receptor; SHP: Small heterodimer partner; CYP7A1: Cholesterol 7α-hydroxylase; CYP27A1: Cholesterol 27α-hydroxylase; BA: Bile acid; Npc1L1: Niemann-Pick C1-like 1; ABCG5: ATP-binding cassette transporters G5; ABCG8: ATP-binding cassette transporters G8; TICE: Transintestinal cholesterol excretion; Ly6C: Lymphocyte antigen 6 complex locus C; ATG16L1: Autophagy related 16 like 1; ET-1: Endothelin 1; Ca^2+^: Calcium; ZO-2: Zonula occludens 2; VE-cadherin: Vascular endothelial cadherin; CROT: Carnitine-O-octanoyl transferase; TNAP: Tissue non-specific alkaline phosphatase; Runx2: Runt-related transcription factor 2; BMP2: Bone morphogenetic protein 2; BMSC: Bone marrow-derived mesenchymal stem cells.

Additionally, TMAO increases tissue factor (TF) production, which promotes thrombogenic activity and vascular senescence characterized by decreased cell migration and proliferation, while simultaneously increasing cell permeability by decreasing levels of the zonula occludens-2 (ZO-2), occluding, and vascular endothelial cadherin (VE-cadherin) proteins via the aforementioned inflammatory pathways [50]. The trace amine-associated receptor 5 (TAAR5) is known to be a specific TMA-only receptor [12]. In addition, TMAO binds directly to protein kinase R-like endoplasmic reticulum kinase (PERK), a crucial signaling pathway that prepares cells for ERS, leading to an increase in fork-head box protein 01 (Fox01) and glucose-regulated protein 97 (GRP-97), important transcription factors involved in insulin signaling and altered glycemic control [51].

**Table 1:** Relationship between atherosclerosis, cardiovascular disease, and TMAO in different experimental models

**Species/Cells**	**Alterations of TMAO levels, consequences, and remarks with proposed mechanisms**	**References**
Mice, HAECs, and VSMC	Increased TMAO levels related to:– increased expression of pro-inflammatory cytokines through MAPK/NF-κB pathway,– increased leukocyte adhesion to the endothelial wall.	[2]
HUVECs	Increased TMAO induced inflammation and endothelial dysfunction through the ROS-TXNIP-NLRP3 inflammasome pathway.	[3]
HUVECs	Increased TMAO levels related to:– increased endothelial dysfunction,– decreased endothelial self-repairing ability,– increased adhesion of monocytes through activation of NF-κB/PKC/VCAM-1 pathway.	[4]
Mice and HUVECs	TMAO induced vascular inflammation through activation of NLRP3 inflammasome through the SIRT3-SOD2-mtROS signaling pathway.	[5]
HUVECs and VSMC	TMAO induced inflammation by stimulating ROS in VSMCs and HUVEC through the AMPK-SIRT1 pathway. TMAO induced increased levels of IL-1, IL-6, TNF-α, NF-κB, MMP9, and NLRP3 in VSMCs and HUVEC. TMAO decreased SIRT1 in VSMCs and HUVEC that is dependent on AMPK activity.	[31]
Mice	Increased TMAO enhanced CD36 expression and foam cell formation through ox-LDL. Foam cell formation was attenuated using siRNA-mediated knockdown of CD36.	[31, 32]
Rats	Increased TMAO related to:– enhanced oxidative stress leading to endothelial dysfunction and vascular inflammation,– increased expression of pro-inflammatory cytokines,– decreased eNOS expression (corrected with DMB).Circulating TMAO levels were increasing with age.	[34]
Murine macrophage J774A.1 cells	Increased TMAO levels related to:– increased expression of SR-A1 (pro-atherogenic),– increased ERS,– decreased ATP-binding cassette (ABC) transporter A1.	[47]
Mice	Increased TMAO enhanced the expression of Fox01.TMAO selectively activated PERK to induce atherosclerosis.	[51]
Mice	Increased TMAO related to:– higher expression of macrophages scavenger receptors, CD36, and SR-A1,– increased lipid accumulation and foam cell formation.	[54]
Human	Elevated levels of TMAO were associated with a higher risk of MACE.	[54, 55]
Human platelet cells	TMAO promoted platelet hyperresponsiveness by enhancing the stimulus-dependent release of intracellular Ca_2_^+^.	[55]
Mice	TMAO induced atherosclerosis through the CD36-dependent MAPK/JNK pathway.	[56]
Mice	Increased TMAO levels related to:– increased expression of pro-inflammatory cytokines,– increased expression of TNF-α,– increased expression of IL-1β,– decreased expression of anti-inflammatory cytokines (IL-10).	[57]
Mice and HUVECs	TMAO accelerated in vivo and in vitro VEC pyroptosis.TMAO induced mitochondrial damage and promoted ROS production.TMAO upregulated SDHB expression of VECs in vitro and in vivo that is involved in TMAO-induced atherosclerosis.	[58]
Human and mice	Increased TMAO related to higher lipid accumulation and alters cholesterol and sterol metabolism.	[59]
Human	Increased TMAO levels related to prevalent CVD.	[60]
Human	Patients after bariatric surgery showed increased plasma TMAO concentration.	[61]
Human	Short and long-term increased TMAO levels in patients after bariatric surgery	[62]
Human and mice	TMAO altered cholesterol and sterol metabolism in distinct compartments.TMAO lowered the expression of CYP7A1, the main bile acid synthetic enzyme.Increased TMAO levels promoted suppression of the RCT.TMAO reduced the expression of intestinal cholesterol transporters Npc1L1.	[44, 63]
Human	A positive correlation between endothelial dysfunction and inflammatory biomarkers with increased TMAO.	[64]
Human	TMAO was correlated with increased ADMA as a marker of endothelial dysfunction in patients with DMT2.	[65]
Human	High levels of betaine were associated with CVD risk in diabetic patients.	[66]
Human	Supplementation with L-carnitine seemed to improve some features of CVD although it raises plasma TMAO and TMA levels.	[67]
Mice	TMAO showed positive effects against atherosclerosis in ApoE^-/-^ transgenic mice.	[68]
Human	Increased TMAO levels related to:– increased cases of cardiac failure,– increased diastolic dysfunction.It was uncorrelated with markers of inflammation.	[69]
Mice	Increased TMAO levels related to:– increased platelet responsiveness to multiple agonists,– increased platelet adhesion to the matrix in whole blood, but does not impact tail bleeding time,– increased thrombosis potential.	[70]
Mice	FMO3 expression and TMAO levels in mice have been shown to exhibit a strong gender bias, and testosterone inhibition of FMO3 expression is shown to underlie the gender difference.FXR expression is an important regulator of FMO3.	[71]
Human	Increased TMAO levels were associated with increased vulnerability of atherosclerotic plaque characteristics in CAD patients, observed with OCT.	[72]
Human	A short-term high-fat diet did not increase fasting plasma TMAO concentrations but it did increase post-prandial TMAO concentrations in healthy non-obese young males.	[73]
Human	TMAO showed a U-shaped association with mortality in elderly patients with acute VTE.	[74]
Human	Long-term increases in plasma TMAO levels were associated with the CHD incidence.Adherence to healthy dietary patterns may modulate the adverse relationship between TMAO changes and CHD.	[75]
Human	TMAO levels correlated with the vulnerable development of coronary plaque and plaque rupture assessed by OCT.	[76]
Human	TMAO was significantly correlated with the incidence of calcification in the culprit lesion segment assessed using OCT in STEMI patients.	[77]
Mice and HUVECs	TMAO accelerated endothelial cell senescence and vascular aging through oxidative stress by suppressing SIRT1 expression.	[78, 79]
Human	TMAO increased risk of CVD through increasing atherosclerosis and calcification process.	[80, 81]
Human	Plasma TMAO level above 6 µM was an independent risk factor for developing CAD incident.	[82, 83]


This table presents experimental studies highlighting the correlation between TMAO and different initial mechanisms of cardiovascular disease, including atherosclerotic plaque formation and instability. TMAO: Trimethylamine-N-oxide; HUVECs: Human umbilical vein endothelial cells; NF-κB: Nuclear factor kappa light chain enhancer of activated B cells; PKC: Protein kinase C; VCAM-1: Vascular cell adhesion molecule-1; HAECs: Human aortic endothelial cells; VSMC: Vascular smooth muscle cells; MAPK: Mitogen-activated protein kinase; TNF: Tumor necrosis factor; IL: Interleukin; CD: Cluster of differentiation; SR-A1: Scavenger receptor A1; CVD: Cardiovascular disease; CYP7A1: Cholesterol 7 α-hydroxylase; RCT: Reverse cholesterol transport; Npc1L1: Niemann-Pick C1-like1; ox-LDL: Oxidized low-density lipoprotein; siRNA: Small interfering RNA; eNOS: Endothelial-derived nitric oxide synthase; DMB: 3,3-dimethyl-1-butanol; MACE: Major adverse cardiovascular events; ADMA: Asymmetric dimethylarginine; DMT2: Diabetes mellitus type-2; TMA: Trimethylamine; ApoE: Apolipoprotein E; ERS: Endoplasmic reticulum stress; ATP: Adenosine triphosphate; FMO3: Flavin-containing monooxygenase-3; FXR: Farnesoid-X-receptor; OCT: Optical coherence tomography; VTE: Venous thromboembolism; ROS: Reactive oxygen species; TXNIP: Thioredoxin interacting protein; NLRP3: Nucleotide oligomerization domain (NOD)-like receptors 3; CHD: Chronic heart disease; SIRT3: Sirtuin 3; SOD2: Superoxide dismutase-2; mtROS: Mitochondrial reactive oxygen species; STEMI: ST elevation myocardial infarction; AMPK: Adenosine monophosphate (AMP)-activated protein kinase; SIRT1: Sirtuin 1; MMP9: Matrix metallopeptidase 9; CAD: Coronary artery disease; Fox01: Fork-head box protein 01; PERK: Protein kinase R-like endoplasmic reticulum kinase; JNK: C-jun N-terminal kinase; SDHB: Succinate dehydrogenase complex iron-sulfur subunit B; VECs: Vascular endothelial cells.

The process of how TMA/TMAO affects atherosclerosis is shown in Figure 2, and detailed information on various studies regarding the effects of TMA/TMAO on CVD is shown in Table 1. TMAO increases platelet hyperreactivity by altering the platelet calcium (Ca^2+^) signaling, which promotes stimulus-dependent platelet activation and increases the responsiveness of cells to agonists, such as thrombin, adenosine 5’-diphosphate (ADP), and collagen, when stimulated, preceding the cascade response leading to platelet aggregation and thrombus formation [5]. Importantly, even in participants receiving low doses of aspirin, higher levels of TMAO were associated with increased platelet aggregation response in a dose-dependent manner, suggesting the possibility that TMAO is involved in platelet reactivity during aspirin treatment and in the so-called aspirin resistance [52]. Moreover, as TMAO levels rise, the vascular endothelium expresses TF, the catalyst for extrinsic coagulation, leading to the pronounced inflammatory TF-dependent prothrombotic effect. Several animal studies using mouse models explain that TMAO is involved in platelet reactivity through the TF pathway, which contributes to major adverse cardiovascular events (MACE). Another in vivo study with mice shows that acute TMAO exposure leads to a significant increase in TF protein in aortic tissue. Some in vitro studies with the human microvascular endothelial cell line 1 (HMEC-1) show that the physiological amount of TMAO is sufficient to induce TF expression. This suggests that an increase in TMAO levels induces vascular TF, possibly leading to vascular thrombosis. Further studies remain to be performed. These have shown that TMAO plays an essential role in modifying thrombogenic activity [53].

## TMA/TMAO formation inhibitors

### 3,3-Dimethyl-1-butanol (DMB)

DMB, a non-toxic structural analog of choline as a specific microbial cutC/D and yeaW/X TMA lyase inhibitor, reduces TMA formation through two main methods: 1) direct suppression of microbial choline TMA lyase activity and 2) reduction of the number of TMA-producing gut microbes. These results are of great importance for the development of novel drugs for the prevention of atherosclerosis in patients, which significantly reduce TMAO levels and promote the reorganization of the GM without significantly reducing the number of beneficial bacteria [54, 84]. DMB provides a structure that can bind competitively but not demonstrate reactivity, preventing choline from being cleaved to TMA. DMB also prevents the conversion of choline, carnitine, and crotonobetaine to TMA (but not GBB), consistent with the finding that DMB has no inhibitory effect on cleavage catalyzed by cntA/B. DMB did not affect the ability of live bacteria to absorb choline demonstrating that it does not prevent choline from entering cells. Balsamic vinegar, red wine, grape seed oil, and extra virgin cold-pressed olive oil are dietary sources of DMB [54].

Although blood cholesterol levels did not change appreciably, DMB prevented the progression and instability of atherosclerosis plaques by reducing plasma TMAO levels, concurrently resulting in a reduced amount of cholesterol accumulation in the macrophages, reduced formation of macrophage foam cells and decreased platelet reactivity [85]. One research found that DMB injection reduced the activation of the thrombosis process while attenuating the increase in platelet reactivity and the in vivo rate of thrombus formation. In platelet-rich plasma (PRP) obtained from choline-treated mice, DMB significantly decreased TMAO levels as well as ADP stimulus-dependent platelet aggregation [86].

Elevated TMAO levels have been shown to promote the development of inflammatory cytokines, whereas DMB has been shown to reverse this tendency by blocking several aforementioned inflammatory signaling pathways. All these mechanisms slow the growth of unstable or newly developing atherosclerotic plaques [87]. In conclusion, inhibition of TMA lyase may lower plasma TMAO levels and protect against atherosclerosis. With these potent pharmacological tools at our disposal, it is now conceivable to transition TMAO-lowering drugs from preclinical models to human research.

### Indole-3-carbinol (I3C)

Recent studies have started to illuminate the significance of the key hepatic drug-metabolizing oxidative enzymes, FMOs, which are found in the ER and can oxygenate a wide range of soft nucleophilic xenobiotics. FMOs occur in a family of five isoforms, including a pseudogene for a sixth FMO. FMO3, followed by FMO1, are the most important FMOs in the TMA metabolism. In the active site of FMOs, TMA interacts with the intermediates nicotinamide adenine dinucleotide phosphate (NADP+) and activates flavin-4-hydroperoxide (FAD-OOH) [16].

Glucobrassicin, a compound found in cruciferous vegetables, such as broccoli, kale, and Brussels sprouts, is hydrolyzed to I3C, a dietary AhR ligand precursor important in the regulation of the immune response. Clinical studies with this compound have demonstrated its effectiveness in reducing the relative ratio of TMAO to TMA by inhibiting FMO3, further reducing atherosclerotic plaque [87].

Pretreatment with I3C in a mouse model of myocardial ischemia/reperfusion injury (MIRI) reduced the MIRI-induced infarct size, serum creatine kinase (CK), and lactate dehydrogenase (LDH). In addition, pretreatment with I3C increased antioxidant parameter levels while decreasing oxidative stress parameters and inflammatory cytokines in MIRI-induced ischemic heart tissue. These results indicate that the anti-apoptotic, antioxidant, and anti-inflammatory properties of I3C treatment protect the heart from MIRI [88].

On the other hand, dietary indoles have an inherent problem with specificity because they are effective serotonin agonists that can significantly penetrate the blood–brain barrier and cause potentially harmful psychotropic effects. The inhibition of FMO enzymes additionally may not be the best option since it leads to accumulation of TMA, which can promote inflammation and the “fishy odor syndrome”. The potentiation of liver damage in diabetic rats caused by elevated cytochrome P450 family 2E1 enzyme (CYP2E1) is demonstrated by inhibition of FMO1 with I3C [89]. Given that FMOs are important enzymes of xenobiotic metabolism, the adverse effects of their inhibition should also be considered. Combination with other TMAO inhibitors, such as DMB, may reduce the need for full-dose I3C use, thus preventing its side effects from becoming significant while effectively maintaining plasma TMAO levels.

### Iodomethylcholine (IMC) and fluoromethylcholine (FMC)

IMC and FMC are analogs of choline and exert an inhibitory effect on the TMA lyase. They were first developed in the study by Roberts et al. [86]. Unlike antibiotics, IMC and FMC were specifically designed to be non-lethal, so the effects of microbial resistance are minimal. The key features of IMC and FMC are: they are non-reactive and transportable into the intact intestinal microbiota, non-lethal to the microbes and have an irreversible inhibitor activity on choline TMA lyase, when cleaved [86].

IMC and FMC are the latest inhibitors of TMAO formation that have been developed. They are the second generation microbial cutC/D inhibitors with better specificity, potency, and non-lethal broad activity against a wide range of commensal microbes. IMC and FMC act as choline analog suicide substrate inhibitors [86]. In vitro kinetic studies showed that they function as irreversible competitive inhibitors that are not lethal to the affected microbes [86]. In vivo studies on animal models showed that IMC and FMC exhibited dose-dependent suppressive activity in both luminal TMA and plasma TMAO with no sign of host toxicity [86]. The orally administered inhibitor remained in the gut and exceeded the dose required to inhibit choline TMA lyase. The researchers noted that the inhibitor was transportable through the internal transporter of the substrate, limiting potential toxicities to the host. When combined with a choline diet, the inhibitor could decrease serum TMAO levels, platelet adhesion, and aggregation and carotid artery thrombus formation. In addition, neither inhibitor affected basic host platelet and coagulation functions, making them safer than other antiplatelet medications such as P2Y12 inhibitors and with a lower risk of bleeding [90].

The gut microbiota of mice showed community-level changes upon IMC and FMC exposure, such as an increase in taxa associated with *Akkermansia* species, which has a beneficial effect by producing mucus from host usable energy sources through anaerobic fermentation [91, 92]. Although IMC was developed as a prototype in this inhibitor model, FMC showed a stronger effect than IMC. Its fluorine substitution is beneficial in the chemistry of suicide substrate inhibitors and its potential use as medication [93].

### Trigonelline

Trigonelline is derived from the hydroalcoholic extract of the plant *Trigonella foenum-graecum*. This plant has been shown to be antihyperlipidemic, antihyperglycemic, and antioxidant in experimental models. It has antibacterial activity against various bacteria: *Escherichia coli*, *Pseudomonas aeruginosa*, *Proteus mirabilis*, *Klebsiella pneumonia*, *Acinetobacter baumanni*, *Staphylococcus aureus*, *Enterococcus faecalis*, and *Bacillus subtilis* [94].

In an in vitro culture, 30 anaerobic microbes were involved in TMA production and *Citrobacter freundii* was superior among them [94]. There was a significant reduction in TMA and TMAO production in the in vitro culture of *C. freundii* enriched with choline and supplemented with trigonelline. In ex vivo, a reduction of a maximum of 85.3% TMAO production by trigonelline was examined at a concentration of about 300 µg/mL [95]. Trigonelline exerted this ability due to its inhibition of the enzyme FMO3. In addition, trigonelline was able to lower the serum levels of triglyceride, TC, LDLs, and glucose in the pathogenic choline diet group through a mechanism that has not yet been explained [95]. This may raise the possibility that trigonelline could prevent the occurrence and development of choline diet-induced hypercholesterolemia [96].

### Potential combination therapy

FMO3 inhibitors have a strong effect and their use is potentially useful in decreasing TMAO formation. However, this also raises problems because excessive inhibition of TMAO formation increases TMA concentration (Figure 1) and potentially causes trimethylaminuria (fish odor syndrome) [97]. In addition, FMO3 inhibitors also have hepatotoxic effects [71]. Therefore, the control of TMAO and TMA levels with the lowest dose of FMO3 inhibitors is required to optimize therapeutic effects and avoid adverse events. This can be achieved by combining the use of TMA lyase and FMO3 inhibitors (Figure 1). The combination of TMA lyase and FMO3 inhibitors provides several beneficial effects: (1) it reduces the risk of fish odor syndrome during the use of FMO3 inhibitors, (2) a lower TMA concentration consequently lowers TMAO concentration, thus potentially improving the outcomes, (3) lower doses of FMO3 inhibitor is required to achieve the target TMAO concentration, minimizing the risk of hepatotoxicity, (4) both FMO3 inhibitors and TMA lyase inhibitors have a protective effect against atherosclerosis and plaque rupture, so the use of their combination may lead to a better outcome.

Choline TMA lyase inhibitor is a type of enzyme mainly responsible for TMA formation. Therefore, its inhibitor, such as DMB, is preferable to significantly reduce TMA formation. In addition, I3C could be a candidate for FMO3 inhibitors because of its potent inhibition of TMA/TMAO formation. The combination of IMC/FMC and trigonelline may also be used. However, there is still not sufficient data and studies regarding these medications, which makes it difficult to compare their features to DMB and I3C.

Despite all other alternative interventions that would prevent or reduce the formation of TMAO, the combination of choline TMA lyase and potent FMO3 inhibitors is being considered because it could provide optimal therapeutic effects with fewer side effects in inhibiting atherosclerosis formation and plaque rupture. However, clinical studies are needed to provide strong evidence on this issue.

## Conclusion

There is strong evidence that gut microbiota-derived processes are associated with a wide range of CVD-related phenotypes, including atherosclerosis, platelet reactivity with thrombosis potential, cholesterol transport, lipid metabolism, obesity, foam cell formation, vascular inflammation, and vascular calcification. DMB, IMC, and FMC reduce the production of TMA by inhibiting choline TMA lyase. I3C and trigonelline block TMA oxidation by inhibiting FMO3, resulting in a decrease in plasma TMAO levels. The combination of choline TMA lyase and FMO3 inhibitors may provide a therapeutic approach to prevent CVDs by reducing plaque formation or stabilizing atherosclerotic lesions. Further clinical studies on animals are needed to confirm this approach to cardiovascular disease prevention.
